# The long pentraxin PTX3: a novel serum marker to improve the prediction of osteoporosis and osteoarthritis bone-related phenotypes

**DOI:** 10.1186/s13018-021-02440-3

**Published:** 2021-04-30

**Authors:** Virginia Veronica Visconti, Chiara Greggi, Simona Fittipaldi, Donato Casamassima, Maria Grazia Tallarico, Francesco Romano, Annalisa Botta, Umberto Tarantino

**Affiliations:** 1 Department of Biomedicine and Prevention, Medical Genetics Section, University of Rome “Tor Vergata”, Via Montpellier 1, 00133 Rome, Italy; 2 Department of Orthopaedics and Traumatology, “Policlinico Tor Vergata” Foundation, Viale Oxford 81, 00133 Rome, Italy; 3 Department of Clinical Sciences and Translational Medicine, University of Rome “Tor Vergata”, Via Montpellier 1, Rome, 00133 Italy

**Keywords:** PTX3, Biomarker, Osteoporosis, Osteoarthritis, Bone-related phenotypes, Inflammation

## Abstract

**Background:**

The long pentraxin PTX3 is generating great interest given the recent discovery of its involvement in bone metabolism. This study investigates the role of circulating PTX3 as a marker of bone-related phenotypes in patients with osteoporosis (OP) and osteoarthritis (OA).

**Methods:**

Serum PTX3 levels were determined using an enzyme-linked immunosorbent assay (ELISA) in a total of OP (*n*=32), OA (*n*=19) patients and healthy controls (CTR; *n*=25). ROC curve analysis was carried out to evaluate the potential of PTX3 for the diagnosis of bone-related phenotypes. In addition, the association between PTX3 serum levels and biochemical markers was estimated by Spearman correlation analysis.

**Results:**

Serum analysis reveals a statistically significant increase of PTX3 levels in OP and OA patients, compared to CTR subjects (**** *p* < 0.0001, **** *p* < 0.0001). ROC curve of PTX3 levels exhibits an excellent sensitivity and specificity for OP and OA diseases (**** *p* < 0.0001 and **** *p* < 0.0001, respectively). Moreover, serum PTX3 levels are positively associated with ALP (*r* = − 0.5257, *p* = 0.0083) and PTH levels (*r* = 0.4704, *p* = 0.0203) in OP patients.

**Conclusions:**

These results confirm the pivotal role of PTX3 in bone metabolism and suggest its potential use as a predictor of OP and OA bone-related phenotypes.

**Supplementary Information:**

The online version contains supplementary material available at 10.1186/s13018-021-02440-3.

## Introduction

The long pentraxin PTX3 is a member of pentraxins superfamily, a class of humoral pattern recognition molecules, which play a fundamental role in innate and adaptive immune response [1, 2]. The human gene encoding PTX3 (*PTX3*, *MIM#602492*) is localized on chromosome 3q25 and is organized in three exons and two introns [3]. The mature protein is a complex octameric molecule characterized by a long N-terminal domain and a C-terminal domain homologous to the classical pentraxins family [4]. PTX3 is an acute-phase protein that is rapidly produced and released by different cell types in response to pro-inflammatory stimuli (6). In addition to its key role in early stages of inflammatory processes, a crucial role in the orchestration of tissue remodelling has also been described [5–7]. Although the main roles of PTX3 discovered so far were in host protection, harmful effects have been observed under pathophysiological conditions suggesting its dual role, even as a negative modulator in a mouse model of intestinal ischaemia and reperfusion [8]. In the last few years, several studies focused on the involvement of PTX3 in bone-related pathophysiological processes, such as ectopic calcifications formation and alteration of bone metabolism in ageing-related diseases [9,10–12]. Interestingly, ageing-related diseases are closely related to inflammatory processes, two conditions belonging to the world of “inflammaging”. The inflammaging is a pathological state characterized by chronic, asymptomatic and systemic low-grade inflammation, involved in different bone-related pathologies, such as osteoporosis (OP) and osteoarthritis (OA) [13–15]. In this context, inflammation is characterized by the excessive secretion of cytokines with a pro-inflammatory role and the ageing process presents an imbalance of the immune system, which leads to an upregulation of immune responses [16, 17]. Recently, interesting data are emerging from studies focused on the role of PTX3 in bone metabolism, both in physiological and pathological conditions. In the pathological context, tumour necrosis factor-alpha (TNF-α) leads to increased PTX3 expression in bone tissue, which results in an enhanced release of receptor activator of nuclear factor kappa-B ligand TNF alpha (RANKL) from osteoblast’s precursors, directing their differentiation towards the osteoclastogenic lineage [18]. Moreover, an accumulation of PTX3 seems to occur in the arthritic joint, suggesting its involvement in triggering a local inflammatory process and degeneration of bone tissue [12]. These results indicate that PTX3 can act as an inflammatory mediator in promoting bone loss in inflammatory bone-erosive diseases [18]. Recently, Scimeca and colleagues investigate PTX3 expression and function in human osteoblasts, describing an impaired PTX3 expression in OP and OA patients, respect to young subjects not affected by bone pathologies. A positive correlation among PTX3 expression, bone density and osteoblast proliferation/maturation is reported, indicating that PTX3 could act as a promoter of bone deposition [11]. Accordingly to the role played by PTX3 as a local regulator of bone metabolism, another study demonstrated that PTX3-deficient mice show impaired bone formation during physiological remodelling and regeneration following fracture injury [19]. In order to gain further insights into the function of PTX3 in bone pathophysiology, we analyse the level of circulating PTX3 in two groups of patients with ageing-related bone metabolism disorders. To this purpose, PTX3 serum levels have been determined in OP patients (*n*=32), OA patients (*n*=19) and healthy subjects (*n*=25). Moreover, circulating PTX3 levels have also been correlated with clinical parameters and biochemical markers of bone metabolism in our cohort of patients. The results obtained confirm the pivotal role played by PTX3 in bone homeostasis, identifying this molecule as a potential non-invasive diagnostic marker associated with OP and OA bone-related phenotypes.

## Materials and methods

### Subjects

All experiments described in the present study were approved by the ethics committee of “Policlinico Tor Vergata” (approval reference number #85/12; June 2017). All experimental procedures were carried out according to The Code of Ethics of the World Medical Association (Declaration of Helsinki). Informed consent was obtained from all patients prior to surgery. Specimens were handled and carried out in accordance with the approved guidelines. This study included 76 patients who underwent surgery in the Orthopaedic Department of “Tor Vergata” University Hospital. Subjects were divided into three groups of analysis: 32 osteoporotic patients (OP) who underwent surgery for fragility fractures following low-energy trauma, 19 osteoarthritic patients (OA) who underwent hip arthroplasty and 25 healthy controls (CTRs) who underwent surgery for high-energy fractures. Exclusion criteria were history of cancer, myopathies or other neuromuscular diseases or chronic administration of corticosteroid for autoimmune diseases (more than 1 month), alcohol abuse and HBV, HCV or HIV infections. The main characteristics and comorbidities of OP, OA and CTR individuals are described in Table 1 and Table S1, respectively.

**Table 1:** Demographic and clinical characteristics of OP patients, OA patients and healthy CTRs

	OP (***n***=32)	OA (***n***=19)	CTRs (***n***=25)	***T*** test (Mann-Whitney test)
**Age (years)**	77.65 ± 11.19	71.95 ± 5.80	43.68 ± 13.32	OP vs CTRL **** (*P* < 0.0001); OA vs CTRL **** (*P* < 0.0001)
**BMI (kg/m**^**2**^**)**	25.99 ± 4.61	28.85 ± 5.41	24.17 ± 3.58	OP vs CTRL ns (*P* = 0.370); OA vs CTRL ** (*P* = 0.004)
**BMD L1–L4**	0.94 ± 0.18	1.35 ± 0.19	1.26 ± 0.15	OP vs CTRL **** (*P* < 0.0001); OA vs CTRL ns (*P* = 0.303)
***t*-score L1–L4**	− 1.99 ± 1.37	1.56 ± 1.61	0.78 ± 1.11	OP vs CTRL **** (*P* < 0.0001); OA vs CTRL ns (*P* = 0.290)
**BMD FN**	0.75 ± 0.13	1.02 ± 0.18	0.93 ± 0.09	OP vs CTRL *** (*P* < 0.001); OA vs CTRL ns (*P* = 0.321)
***t*-score FN**	− 2.15 ± 0.80	− 0.02 ± 1.35	− 0.63 ± 0.68	OP vs CTRL **** (*P* < 0.0001); OA vs CTRL ns (*P* = 0.485)
**Calcium (mg/dl)**	8.99 ± 0.44	8.55 ± 0.34	9.27 ± 0.61	OP vs CTRL ns (*P* = 0.186); OA vs CTRL ** (*P* = 0.003)
**Phosphorus (mg/dl)**	3.07 ± 0.68	–	3.27 ± 0.69	OP vs CTRL ns (*P* = 0.419)
**PTH (pg/ml)**	122.38 ± 91.34	84.84 ± 29.63	58.57 ± 24.22	OP vs CTRL ** (*P* = 0.026); OA vs CTRL ns (*P* = 0.061)
**25-(OH)-Vit D (ng/ml)**	15.99 ± 15.28	17.16 ± 7.39	24.81 ± 15.58	OP vs CTRL * (*P* = 0.013); OA vs CTRL ns (*P* = 0.148)
**ALP (U/l)**	80.52 ± 20.79	69.37 ± 16.71	72.33 ± 12.49	OP vs CTRL ns (*P* = 0.275); OA vs CTRL ns (*P* = 0.490)

*BMI* body mass index, *BMD* bone mineral density, *PTH* parathyroid hormone, *25-(OH)-Vit D* 25-hydroxyvitamin D, *ALP* alkaline phosphatase. * *p*<0.05, ** *p*<0.01, *** *p*<0.001, **** *p*<0.0001

### Specimen collection

Whole blood from participants was drawn after overnight fasting and collected in tubes with floating separator gel. Blood samples were centrifuged, within an hour of collection, at 1500×*g* for 20 min at 4 °C. Serum phase was harvest and transferred in RNase-free tubes. Additional centrifugation, at 16,000×*g* for 10 min at 4 °C, to remove residual cells was performed. Serum samples were stored at − 80 °C until further analysis.

### Bone mineral density evaluation and radiographic analysis

Dual-energy X-ray absorptiometry (DXA) was performed with a Lunar DXA apparatus (GE Healthcare, Madison, WI, USA). Lumbar spine (L1–L4) and femoral (neck and total) scans were performed, and bone mineral density (BMD) measurements (g/cm^2^) along with *T*-scores were recorded according to the manufacturer’s recommendations [20]. DXA exam was performed one day before surgery for OA patients, one month after surgery for OP patients, and within a week after surgery for CTR individuals. Hip radiographs of all participants were obtained using a standard and validated protocol [21]. Two orthopedists independently assessed all radiographs using the Kellgren-Lawrence (K-L) radiographic atlas [22].

### Clinical and biochemical parameters

All individuals were subjected to the dosage of bone metabolism markers: calcium, phosphorous, parathyroid hormone (PTH), 25-hydroxyvitamin D (25-(OH)-Vit D) and alkaline phosphatase (ALP). Finally, we collected data regarding age, gender, height, weight, dietary habit, smoke, alcohol intake, personal and familial clinical history. The detailed clinical characteristics of the study subjects are summarized in Table 1.

### Measurement of serum PTX3 concentration

The serum PTX3 concentration was determined using an enzyme-linked immunosorbent assay kit (ab214570 Human Pentraxin 3 ELISA Kit), according to the manufacturer’s protocol. The analysis was performed using serum previously stored frozen at − 80 °C and the PTX3 levels were all measured at the same time using reagents with the same lot numbers to reduce the measurement variability. The standard curve of the assay was constructed by applying a serial dilution. The starting concentration of PTX3 was set to 10,000 pg/ml and from that point diluted 7 times to a final concentration of 15.6 pg/ml. The concentrations of PTX3 were measured in duplicates, interpolated from the PTX3 standard curve, and corrected for sample dilution.

### Statistical analysis

Data were analysed with GraphPad Prism 5.0 (GraphPad Software, Inc., La Jolla, CA, USA). Normality of the distribution of the studied variables was assessed by the Shapiro-Wilk normality test. Non-parametric Mann-Whitney *U* test was used for clinical/biochemical variables. Serum PTX3 levels comparison between groups were carried out using the Kruskal-Wallis test. The optimum cut-off value, sensitivity, specificity and predictive values of PTX3 were determined by the receiver operating characteristic (ROC) analysis with 95% CI. The area under the ROC curve (AUC) of 0.5 to 0.7 is considered low, 0.7 to 0.8 is considered acceptable, 0.8 to 0.9 is considered excellent and more than 0.9 is considered outstanding diagnostic value. The correlation between ELISA and clinical/biochemical data was evaluated using Spearman’s rank test. Data are presented as the mean ± SD. Differences were considered significant when the *p* value was < 0.05 (* *p* < 0.05, ** *p* < 0.01, *** *p* < 0.001, **** *p* < 0.0001).

## Results

### Baseline demographic and clinical characteristics of subjects

We enrolled a total of 76 individuals participating to this study (OP, *n* = 32; OA, *n* = 19; CTR, *n* = 25) between July 2018 and February 2020. The baseline demographic and clinical characteristics are shown in Table 1. The mean age was 78.61 ± 10.29 in OP patients, 72.12 ± 8.75 in OA patients and 44.42 ± 13.58 in healthy subjects. The mean body mass index (BMI) was significantly higher in OA patients with respect to health CTRs (** *p* = 0.004). The OP group included patients with fragility hip fracture and *T*-score ≤ − 2.5, the OA group included patients with radiographic evidence of hip and *T*-score ≥ − 2.5 S.D. and CTR subjects were characterized by a *T*-score ≥ − 1.0 S.D. Analysis of biochemical parameters revealed low calcium levels in the OA group compared to CTRs (** *p* = 0.003). Instead, no statistically significant differences in clinical characteristics between OP and CTR groups were found, except for the mean circulating high levels of PTH, and low levels of 25-(OH)-Vit D in OP patients with respect to CTRs (** *p* = 0.026; * *p* = 0.013). Furthermore, the analysis of comorbidities in our cohort of patients reveals that hypertension is the most common symptom in OP (53.1%) and OA (52.6%) patients, whereas no inflammatory diseases have been reported (Table S1). The use of diuretic drugs and calcium channel blockers are the main therapeutic treatments in OP (46.9% and 34.4%, respectively) and OA (42.1% and 36.8%, respectively) groups (Table S1).

### Serum PTX3 levels in OP and OA patients

**Fig. 1:**
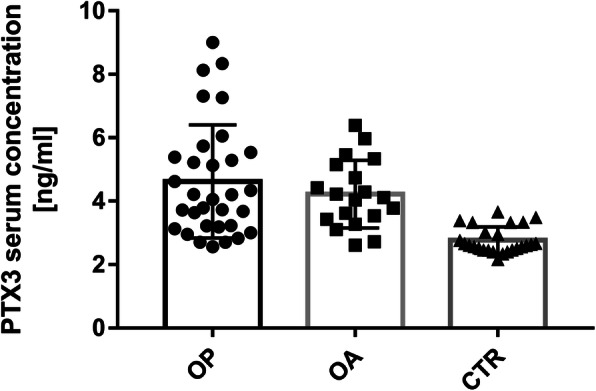
PTX3 serum levels in OP and OA patients compared to CTR subjects. OP and OA patients showed significantly higher mean circulating PTX3 level than CTR subjects (4.62 ± 1.78 ng/ml, 4.22 ± 1.06 ng/ml and 2.77 ± 0.42 ng/ml). **** *P* < 0.0001

PTX3 serum levels in OA, OP and CTR subjects are shown in Fig. 1. Data analysis reveals a statistically significant increase in circulating PTX3 levels in OP and OA patients, compared to CTR subjects (**** *p* < 0.0001, **** *p* < 0.0001). There is no statistically significant difference between OP and OA patients. In detail, PTX3 serum levels in OP patients range from 2.56 to 9 ng/mL, with a mean ± SD of 4.62 ± 1.78 ng/ml and a median value of 4.12 ng/ml. In OA patients, PTX3 serum levels range from 2.61 to 6.39 ng/ml, with a mean ± SD of 4.22 ± 1.06 ng/ml and a median value of 4.11 ng/ml. In the CTR subjects, PTX3 range from 2.16 to 3.66 ng/ml, with a mean ± SD of 2.77 ± 0.42 ng/mL and a median value of 2.61 ng/ml.

### ROC curve analysis

**Fig. 2:**
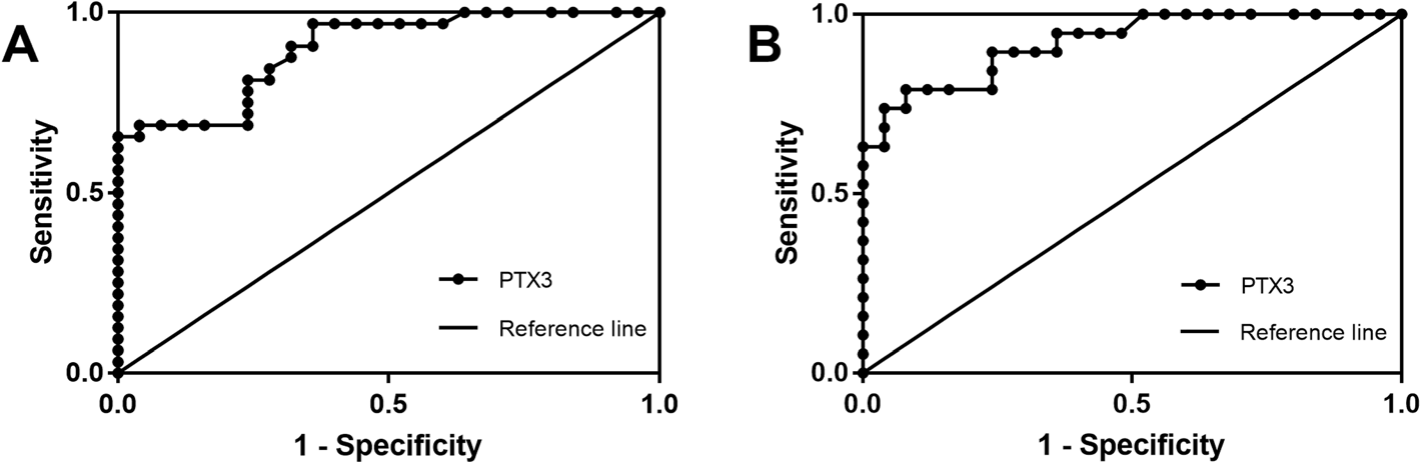
ROC curve analyses of the diagnostic value of serum PTX3 levels. **a** ROC curve of PTX3 levels in OP patients. AUC=0.8988, 95% CI=0.822–0.9755. ****p < 0.0001. **b** ROC curve of PTX3 levels in OA patients. AUC=0.9211, 95% CI=0.843–0.9991. ****p < 0.0001

ROC curve analysis for PTX3 serum levels in OP patients showed that the area under the ROC curve was AUC=0.8988, *p* < 0.0001, 95% CI=0.822–0.9755. When the cut-off value was 2.97, the sensitivity was 84.38% and the specificity was 72% (Fig. 2a). In OA patients, ROC curve analysis for PTX3 serum levels showed that the area under the ROC curve was AUC=0.9211, *p* < 0.0001, 95% CI=0.843–0.9991. When the cut-off value was 3.06, the sensitivity was 89.47% and the specificity was 76% (Fig. 2b). This result suggests that PTX3 in serum can be considered a potential biomarker for the diagnosis of OP and OA bone-related phenotypes.

### Correlation analysis of PTX3 serum levels with clinical/biochemical markers

**Fig. 3:**
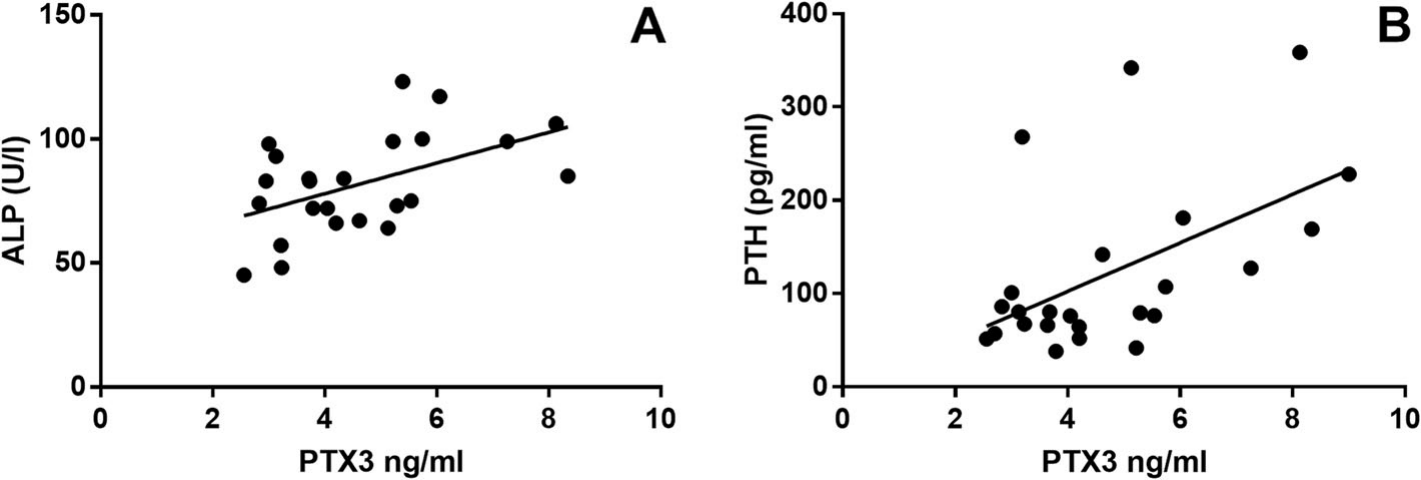
Correlation between serum PTX3 levels and biochemical markers in OP patients. **a** Serum PTX3 level was positively associated with ALP level (U/l). Spearman’s correlation analysis was performed. Spearman *r* = 0.5257; 95% CI = 0.1428 to 0.7717. **P = 0.0083. **b** Serum PTX3 level was positively associated with PTH (pg/ml). Spearman’s correlation analysis was performed. Spearman *r* = 0.4704; 95% CI = 0.07017 to 0.7402. *P = 0.0203

The present study also investigates the correlation between circulating PTX3 levels and clinical/biochemical parameters, including BMI, BMD L1-L4, *t*-score L1-L4, BMD FN, *t*-score FN, calcium, phosphorous, PTH, 25-(OH)-Vit D and ALP in our study cohort. Serum PTX3 levels are positively associated with ALP levels (Fig. 3a, *r* = − 0.5257, *p* = 0.0083) and PTH levels (Fig. 3b, r = 0.4704, *p* = 0.0203) in OP patients. No significant correlations between serum PTX3 levels and the clinical variables of interest were found in OA patients.

## Discussion

The recent discovery of the involvement of PTX3 in bone metabolism [11, 18, 23, 24] highlights the necessity to better investigate its role in ageing-related phenotypes. OP and OA bone-related diseases are highly prevalent world health problems, negatively impacting the quality of life in elderly [25, 26]. Although these are two different diseases, both are characterized by low-grade chronic inflammation promoted by ageing, resulting in elevated cytokine production [14, 27]. PTX3 is released by peripheral blood leukocytes and myeloid dendritic cells in response to primary pro-inflammatory stimuli, such as cytokines, by acting as an essential player in the regulation of inflammation [6, 28]. The involvement of PTX3 in bone metabolism and inflammation in age-related diseases addressed the present study on the analysis of circulating PTX3 levels in OP and OA as paradigms of bone-related phenotypes. There are few and conflicting studies analysing PTX3 expression levels in bone metabolism in pathophysiological conditions. Indeed, two independent studies on human osteoblasts show an opposite regulation of PTX3 expression level in pathological conditions related to bone diseases [11, 18]. However, the positive role of PTX3 has been exalted in a study showing an impaired bone formation during physiological remodelling in a PTX3-deficient mouse model [19]. Unfortunately, to date, the expression level of circulating PTX3 correlated with human bone metabolism was analysed only in a study which reported an inverse association between plasma PTX3 levels and BMD of the lumbar spine and femoral neck in a large cohort of Korean population [24]. Accordingly, our study reveals a relevant increase of PTX3 expression level in serum from OP and OA patients, corroborated by the excellent sensitivity and specificity of the analysed values. Our results suggest an inverse PTX3 regulatory mechanism between cell/tissue and circulatory system. The high serum levels detected in OP and OA patients could be explained by extracellular vesicles (EVs) mechanism, by which cells eliminate unnecessary proteins. Several studies identified an enrichment of PTX3 in EVs correlated with pro-inflammatory conditions during the progression of different cancer types [29–31]. Moreover, the existence of a ready-made form of stored PTX3 in neutrophil-specific granules has also been demonstrated. After activation of these cells by appropriate stimuli, this preformed amount of PTX3 is released into the extracellular space, localizing in neutrophils extracellular traps (NETs) [32, 33]. EVs and NETs mechanisms could explain PTX3 expression levels variability between cell and serum systems, suggesting its dual role as a positive or negative bone-diseases regulator depending on tissue location. In addition, it has been recently shown that epigenetic control plays an important role in modulating PTX3 expression by differential methylation of the promoter and enhancer regions in physiological and inflammatory conditions [34]. Further investigations on the role of PTX3 in OP and OA bone-related phenotypes are therefore needed to better elucidate the detailed mechanisms regulating its system-dependent behaviour. In our study, Spearman correlation analysis reveals a positive association of PTX3 serum levels with ALP and PTH levels in OP patients. The analysis of enrolled patients’ comorbidities excluded that circulating PTX3 levels could be modulated by an inflammatory component. Elevated ALP levels have been detected in postmenopausal OP women and OP men, and it has been proposed that serum ALP levels could be used as an index of decreased bone mineral density [35–37]. In addition, an increased expression of PTH levels causes an up-regulation of RANKL, which in turn leads to an unbalance of remodelling processes and bone resorption, a condition that predisposes to OP development [38–40]. Thus, the positive correlation between the increase in PTX3 levels, with both ALP and PTH bone markers, strengthens the proposed role of PTX3 as a systemic indicator of the pathological state associated with OP disease.

## Conclusions

Our pilot study strongly suggests that PTX3 could be a promising non-invasive biomarker for OP and OA bone-related phenotypes. Although our results are supported by a strong statistical significance, the relatively small number of individuals analysed implies the necessity of additional studies on larger study cohorts. A potential limitation of this study is the lower mean age of control compared to the OP and OA patient groups. However, this potential bias is not easily resolvable because of the difficulty in recruiting elderly people not affected by chronic bone diseases. Further functional investigations are needed to better understand the potential associated with using PTX3 in daily practice as a pathological hallmark of bone-related phenotypes. A novel disease-biomarker to improve the prediction of OP and OA diseases could significantly contribute to the reduction of costs involved in diagnostic procedures and treatment of bone metabolism disorders.

## Supplementary Information

**Additional file 1: Table S1**. Comorbidities and Medical treatments for OP, OA and CTR subjects.

## Data Availability

All data used and analysed during this study are available from the corresponding author.

## Abbreviations

OP: Osteoporosis
OA: Osteoarthritis
CTR: Control
ELISA: Enzyme-linked immunosorbent assay
ALP: Alkaline phosphatase
TNF-α: Tumour necrosis factor-alpha
RANKL: Receptor activator of nuclear factor kappa-B ligand
BMI: Body mass index
BMD: Bone mineral density
PTH: Parathyroid hormone
25-(OH)-Vit D: 25-hydroxyvitamin D
DXA: Dual-energy X-ray absorptiometry
ROC: Receiver operation characteristic
EVs: Extracellular vesicles
NETs: Neutrophils extracellular traps
