# Correction to: The long pentraxin PTX3: a novel serum marker to improve the prediction of osteoporosis and osteoarthritis bonerelated phenotypes

**DOI:** 10.1186/s13018-021-02481-8

**Published:** 2021-05-21

**Authors:** Virginia Veronica Visconti, Chiara Greggi, Simona Fittipaldi, Donato Casamassima, Mariagrazia Tallarico, Francesco Romano, Annalisa Botta, Umberto Tarantino

**Affiliations:** 1 Department of Biomedicine and Prevention, Medical Genetics Section, University of Rome “Tor Vergata”, Via Montpellier 1, 00133 Rome, Italy; 2 Department of Orthopaedics and Traumatology, “Policlinico Tor Vergata” Foundation, Viale Oxford 81, 00133 Rome, Italy; 3 Department of Clinical Sciences and Translational Medicine, University of Rome “Tor Vergata, Via Montpellier 1, 00133 Rome, Italy

**Correction to:** ***J Orthop Surg Res*** **16, 288 (2021)**

**https://doi.org/10.1186/s13018-021-02440-3**

Following the publication of the original article [1], the authors noticed that the given name of one of the authors has been captured incorrectly. Maria Grazia Tallarico should be Mariagrazia Tallarico. The correct name is shown in the author group section above.

The original article has been corrected.

## References

[1] Visconti VV, Greggi C, Fittipaldi S, Casamassima D, Tallarico MG, Romano F (2021). The long pentraxin PTX3: a novel serum marker to improve the prediction of osteoporosis and osteoarthritis bone-related phenotypes. J Orthop Surg Res.

