# Supplementary material for: The long pentraxin PTX3: a novel serum marker to improve the prediction of osteoporosis and osteoarthritis bone-related phenotypes
Source: J Orthop Surg Res. 2021 Apr 30;16:288. doi: 10.1186/s13018-021-02440-3 (PMC8086331; doi:10.1186/s13018-021-02440-3)
Supplement: Supplementary file 1 — Additional file 1: Table S1. Comorbidities and Medical treatments for OP, OA and CTR subjects. [file 13018_2021_2440_MOESM1_ESM.docx]

|  |  |  |  |  |
| --- | --- | --- | --- | --- |
|  |  | **Subjects** | | |
|  |  | **OP** | **OA** | **CTR** |
| **Comorbities** | |  |  |  |
| Hypertension | | 17 (53.1%) | 10 (52.6%) | 4 (16%) |
| Stroke | | 2 (6.2%) | 1 (5.2%) | na |
| Dyslipidemia | | 1 (3.1%) | 2 (10.5%) | na |
| Diabetes mellitus | | 4 (12.5%) | 2 (10.5%) | na |
| Hypercholesterolemia | | 3 (9.4%) | 1 (5.2%) | 5 (20%) |
| Parkinson's disease | | 2 (6.2%) | na | na |
| Alzheimer’s disease | | 2 (6.2%) | na | na |
| Gastroesophageal reflux disease | | 2 (6.2%) | 1 (5.2%) | na |
| Asthma | | 3 (9.4%) | 2 (10.5%) | 1 (4%) |
|  |  |  |  |  |
| **Medical treatments** | |  |  |  |
| Diuretics | | 15 (46.9%) | 8 (42.1%) | 3 (12%) |
| Calcium channel blockers | | 11 (34.4%) | 7 (36.8%) | na |
| Atorvastatin | | 2 (6.2%) | 1 (5.2%) | 3 (12%) |
| Antidiabetic therapy | | 4 (12.5%) | 2 (10.5%) | na |
| Dopaminergic agents | | 1 (3.1%) | na | na |
| Cholinesterase inhibitors | | 2 (6.2%) | na | na |
| Gastroprotective agents | | 1 (3.1%) | 1 (5.2%) | na |
| Corticosteroids | | 2 (6.2%) | 1 (5.2%) | 2 (8%) |

**Table S1. Comorbidities and Medical treatments for OP, OA and CTR subjects.**
